# Identifying the abiotic factors that determine the inland range limits of a mesic-adapted lizard species

**DOI:** 10.1093/icb/icad124

**Published:** 2023-10-19

**Authors:** Jules E Farquhar, Wyn Russell, David G Chapple

**Affiliations:** School of Biological Sciences, Monash University, Clayton, Victoria 3800, Australia; Biosis Pty Ltd, Port Melbourne, Melbourne, Victoria 3207, Australia; School of Biological Sciences, Monash University, Clayton, Victoria 3800, Australia

## Abstract

For most species, the factors that determine geographical range limits are unknown. In mesic-adapted species, populations occurring near the edge of the species’ distribution provide ideal study systems to investigate what limits distributional ranges. We aimed to identify the abiotic constraints that preclude an east-Australian mesic-adapted lizard (*Lampropholis delicata*) from occupying arid environments. We performed lizard surveys at sites spanning an elevation/aridity gradient (380–1070 m) and measured the prevalence of habitat features (logs, rocks, leaf litter, bare ground, solar radiation) in addition to hourly temperatures in a variety of microhabitats available to lizards. Species distribution models (SDM) were used to identify the macroclimatic variables limiting the species’ distribution. At its inland range limit, *L. delicata* is associated with mesic high-elevation forests with complex microhabitat structures, which gradually decline in availability toward lower (and more arid) elevations where the species is absent. Moreover, *L. delicata* is absent from sites with a shallow leaf litter layer, in which daily temperatures exceed the species’ thermal preference range, which we determined in a laboratory thermal gradient. In regards to macroclimate, SDM revealed that temperature seasonality is the primary variable predicting the species’ distribution, suggesting that *L. delicata* avoids inland areas owing to their high annual thermal variability. By combining multiple lines of evidence, this research highlights that habitat and microclimate suitability—not solely macroclimate suitability—are important range-limiting factors for mesic ectotherms and should be incorporated in studies addressing range-limiting hypotheses.

## Introduction

A long-standing objective of biogeography, ecology, and related disciplines is to understand the cause of species’ range limits (von Humboldt and Bonpland 1805; Darwin 1859; Andrewartha and Birch 1954; MacArthur 1972; Sexton et al. 2009). Climate is a key determinant of species distributions because factors such as air temperature and rainfall strongly affect vital physiological functions (Deutsch et al. 2008; Kearney and Porter 2009; Sheldon and Tewksbury 2014; Chown and Gaston 2016). Climate naturally varies over space and time, and populations persist under this variability by altering their physiology through phenotypic plasticity or by adaptation to match the climates they encounter (Holt 1990; Llewelyn et al. 2018). In many cases, however, rates of adaptive evolution lag far behind rates of climatic change (Martinez-Meyer and Peterson 2006; Sinervo et al. 2010; Ordonez 2013; Quintero and Wiens 2013), and lineages therefore tend to retain ancestral traits despite considerable changes in climate (Wiens and Graham 2005; Wiens et al. 2010). An important corollary of this trait conservatism is that populations facing periods of severe climatic changes (e.g., Pleistocene glacial cycles) will undergo vicariance, as the species’ wider distribution becomes uninhabitable, and individuals must migrate to refugial habitats (Wiens and Graham 2005; Byrne 2008; Hua and Wiens 2013). By investigating the range limits of species confined to these refugia, we can identify the abiotic factors that prevent expansion from such areas.

At a species’ range limit, populations will fail to establish in areas where fitness falls below that required for populations to maintain positive growth rates (Holt 2003; Holt et al. 2005; Bridle and Vines 2007; Angert 2009). These unsuitable areas are situated outside of the species’ ecological niche (*sensu*Hutchinson 1957; Soberón 2007), which is the *n*-dimensional set of all biotic and abiotic variables that influence an organism’s ability to survive and reproduce (i.e., its fitness; Holt 2009). There is often a strong relationship between a species’ niche and its distribution; hence, organisms tend to be found in environments that are congruent with their niche properties (James et al. 1984; Pulliam 2000; Kearney 2006; Wiens 2011; Hargreaves et al. 2014; Lee-Yaw et al. 2016). Environmental temperature has been emphasized as a primary niche dimension and thus a range-limiting factor, particularly for ectotherms, because their basic physiological functions are often dependent on the thermal environment (Huey and Stevenson 1979; Huey 1982; Angilletta 2009; Sinervo et al. 2010).

Species distribution models (SDMs) have been widely used to model the relationships between organisms and their environment in order to predict distributions based on important climate-relevant axes of a species’ niche and, in doing so, identify putative range-limiting processes (Kearney and Porter 2004; Kearney and Porter 2009). Mesic refugial areas are known to harbor unusually stable climates that protect organisms from high climatic variability (Byrne 2008), and hence SDMs that incorporate data on this variability can reveal the factors that restrict species to mesic areas. For example, high temperature seasonality has been identified as a key range-limiting factor in frog species (Wiens et al. 2006). While SDMs are useful for understanding such correlative links, they are typically constructed with data derived from satellite macroclimatic measurements and are thus incapable of capturing the buffering microclimatic regimes that are actually experienced by organisms (Scheffers et al. 2014). Microhabitat structures and the microclimates they offer are known to play a vital role in moderating the physiological impacts of macroclimate on ectotherms (Huey and Tewksbury 2009; Scheffers et al. 2014; Sunday et al. 2014). Range limits, then, may emerge because of micro (not strictly macro) climatic unsuitability. As such, empirically measuring (e.g., with temperature data loggers) variation in microclimatic temperatures offers a more holistic approach to exploring the ecophysiological basis of range limits in thermally sensitive species.

Ectotherm activity rates tend to be maximized within a set-point temperature range (*T*_set_; Hertz et al. 1993; Gunderson and Leal 2015), that is, the central 50% of temperatures selected by individuals in a thermal gradient. This is considered the target (or preferred) body temperature range organisms seek to maintain for optimal physiological and ecological performance. Some models of ectotherm thermoregulation consider that activity will be substantially reduced when environmental temperatures exceed a species’ *T*_set_ (Sinervo et al. 2010; Gunderson and Leal 2015). If lizards cannot access microclimates that offer temperatures within or below their *T*_set_, even beneath buffering structures such as leaf litter, then range limits may emerge as the positions in the landscape beyond which effective thermoregulation and activity are unattainable or severely reduced during times of thermal stress.

**Figure 1.**
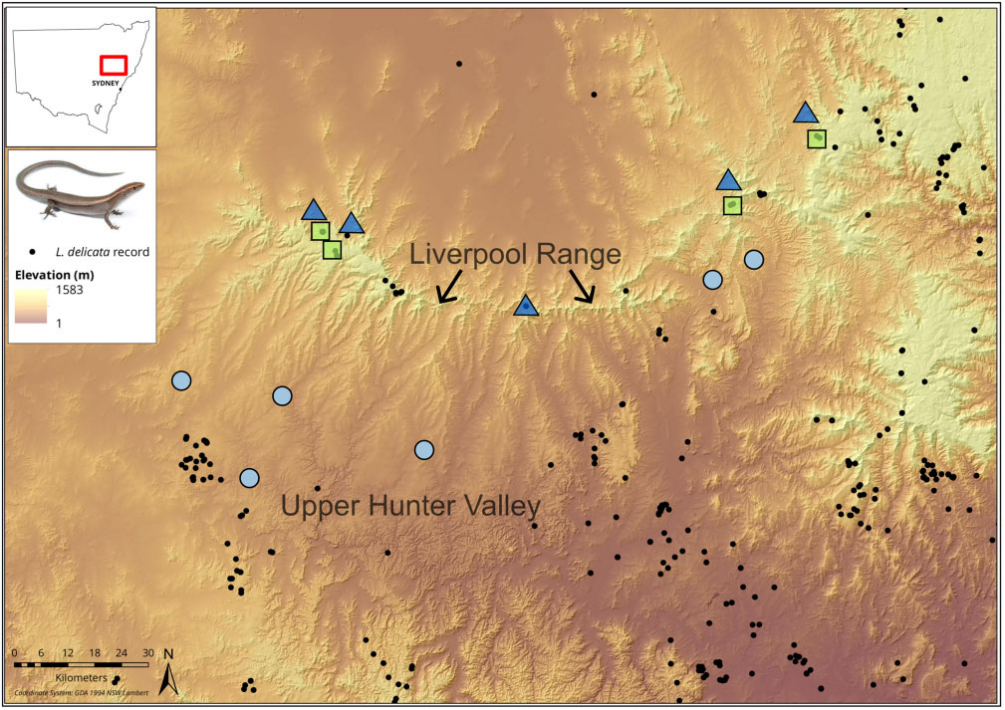
Survey sites and occurrence records of *L. delicata* across the Upper Hunter Valley region. Black points are records obtained from the Atlas of Living Australia (2022). Color symbols denote survey sites in three broad elevational categories: green squares, 970–1070 m (high-elevation sites); dark blue triangles, 780–840 m (mid-elevation sites); and light blue circles, 380–660 m (low-elevation sites). The inset map shows the location of the study region within NSW.

In the present study, we explore the factors that influence the range limits of a widespread, mesic-adapted reptile species, the delicate skink (*Lampropholis delicata*). This species is a small (adult snout-vent length 34–55 mm), diurnal, heliothermic lizard that inhabits moist habitats of south-eastern Australia (Crome 1981; Wilson and Swan 2021). Phylogeographic structuring across the species’ range suggests that populations have contracted to mesic refugial habitats during the early to mid Pliocene expansion of arid habitats, with several genetically distinct lineages separated by major dry habitat barriers, such as the Hunter Valley (Chapple et al. 2011; Fig. 1). The occurrence of disjunctions over dry habitat barriers suggests that temperature and precipitation are potentially important components of the climatic niche in *L. delicata* (Quintero and Wiens 2013; Wiens et al. 2013). Drier inland environments may represent barriers to dispersal for *L. delicata* because they impose high physiological demands that are beyond the limits of the species’ capacity to adapt. Moreover, as microhabitat structures (and the microclimates they offer) are known to play a vital role in moderating the physiological impacts of macroclimate in ectotherms (Huey and Tewksbury 2009; Scheffers et al. 2014; Sunday et al. 2014), spatial differences in these resources are likely important for the species’ persistence. Yet, no attempts have been made to explain the distribution of *L. delicata* with regard to its ecophysiology and the microclimates individuals experience.

Here, we measured variation in habitat structure and microclimate over an elevational gradient ranging from upland mesic refugial habitats to dry low-elevation habitats. We also measured the *T*_set_ of *L. delicata* in order to understand how environmental temperatures (i.e., air temperatures, *T*_a_) deviate from the species’ preferred temperature in habitats both with and without *L. delicata*. Under the assumption that *L. delicata* is a mesic-adapted species, we expected that, at its inland range limits, it would be confined to the structurally complex mesic plateaux of the Liverpool Range. The species is typically associated with leaf litter, so we expected that this is the primary microhabitat within which thermoregulating skinks can maintain body temperatures within their *T*_set_ during the hottest times of the day. From a macroclimatic perspective, we predicted that climate variables describing stability (such as temperature or precipitation seasonality) would be key determinants of the species’ range limit, given that mesic refugia tend to be climatically stable (Byrne 2008).

## Materials and methods

### Study region

The study area is a large (∼8000 km^2^) section of the Upper Hunter Valley region of New South Wales (NSW, Australia; -31.30–32.18°S, 149.22–151.17°E; Fig. 1). The Liverpool Range is the region’s primary source of topographic complexity and high-rainfall habitats. This east–west mountain range is a 100 km inland projection of the Great Dividing Range, with high-elevation peaks and plateaux (∼1200 m a.s.l.) supporting tall grassy and ferny open forests, tall grass tree shrublands, and western outliers of sub-tropical rainforest (Fisher 1985; Benson et al. 2010). These upland mesic habitats transition into rolling hills with dry, open grassy woodland at low elevations of the Hunter Valley (∼300–400 m a.s.l.), though much of this habitat has been converted to intensive agricultural land (Benson et al. 2010). This broad valley of dry habitat forms a major biogeographic barrier, separating the moist upland forests of the basaltic Liverpool Range in the north from those of the Sydney Basin sandstone escarpment in the south (Di Virgilio et al. 2012; Bryant and Krosch 2016).

### Lizard surveys

Given the deep genetic divergence of the *L. delicata* subclade on the western Liverpool Range (i.e., Coolah Tops; Chapple et al. 2011), disjunctions likely occur between this population and those further east. However, records of *L. delicata* are sparse across the region (Fig. 1), and it is unclear to what extent the Liverpool Range subclade is geographically isolated. Under the hypothesis that abiotic factors (macroclimate, microclimate, and habitat structure) influence the species’ distribution, and given the strong effect of elevation on abiotic variability in the region (Ashcroft and Gollan 2013), we performed occupancy surveys at sites (*n* = 15) spanning an elevational gradient: four high-elevation sites (970–1070 m a.s.l.) where *L. delicata* is abundant; four mid-elevation sites (780–840 m a.s.l.) directly downslope of the high-elevation sites; and six low-elevation sites (380–660 m a.s.l.) in the valley. To determine whether the Liverpool Range acts as a “habitat bridge” between Coolah Tops and the eastern plateaux, one additional site was located at a relatively low point (664 m a.s.l.), halfway along the Liverpool Range, where a single unconfirmed record of the species exists (Fig. 1). Each survey site consisted of four 200m transects (20m apart) walked by a single observer during daylight hours to (1) scan the ground for active lizards and (2) rake leaf litter and flip cover (e.g., logs and rocks) for inactive lizards. Lizards were hand-captured to confirm species identification. Fieldwork was undertaken from mid-September to mid-November 2019, coinciding with the species’ breeding season.

### Quantifying habitat structure

Habitat structure was measured at each lizard capture point by estimating the cover percentage of logs, rocks, and bare ground within a 5 m^2^ quadrat centered on each lizard capture point. The deepest section of leaf litter within each quadrat was recorded in mm. A Canon 5D Mark IV (35 mm frame) digital camera with a Canon 8–15 mm f/4L Fisheye lens was used to take a hemispherical photograph of the canopy (>60 cm) above each lizard capture point. The resulting images were processed with the Gap Light Analyzer software (Frazer et al. 1999) to provide a percentage measure of transmitted solar radiation reaching the ground. For sites without *L. delicata*, the same habitat assessments were performed, but with 5 m^2^ quadrats (*n* = 10) centered on random position along survey transects. Data from quadrats were pooled by site and the average value for each variable was calculated to describe the structure of the site. A total of 255 quadrats were assessed.

A principal component analysis (PCA) was performed with the packages *FactorMineR* (v2.4; Lê et al. 2008) and *factoextra* (v1.0.7; Kassambara and Mundt 2017) to reduce multicollinearity in habitat structure data and to visualize patterns of habitat change across an elevational gradient. PC loadings were inspected and the Kaiser–Guttman criterion (eigenvalue > 1; Jackson 1993) was used to select PCs for subsequent analyses. To test for differences in habitat structure over elevation, a one-way ANOVA was used on PC scores of the first two axes followed by Tukey’s post-hoc tests.

### Quantifying microclimate

We deployed iButton data loggers (*n* = 30; Thermochron DS1921G, Maxim, San Jose, USA) set to record hourly air temperatures (*T*_a_; ± 0.5°C) within microhabitats available to *L. delicata* from October 3rd to October 26th, 2019 (October is a time of year when skinks are highly active and reproductive). The species is diurnal; therefore, only daytime temperatures (0700–1900 h) were considered. At each site (*n* = 15), we deployed four iButtons, one in each of the following positions: **shade** (5 cm above the litter layer inside an open-ended PVC tube beneath a plastic dish to create constant shade), **exposed** (on the soil surface subject to sunlight), **litter** (1 cm above the soil surface at the bottom of the leaf litter layer), and **soil** (5 cm below the soil surface). These four positions were chosen because they are the primary microhabitats accessible to lizards attempting to behaviorally thermoregulate. More details relating to iButton deployment are provided in the Supplementary Information (S1). To compare microclimates against the thermal preference of *L. delicata*, we quantified the thermal preference of 111 individuals collected from the study region (collection and husbandry details are provided in Supplementary Information S3) in a controlled laboratory environment (see section “Measuring thermal preference of *L. delicata*”).

### Collecting occurrence records

We obtained occurrence records of *L. delicata* (De Vis 1888) by downloading data through the Global Biodiversity Information Facility on January 16, 2023 (GBIF.org, 2023). The download settings were set to Australia only, with a coordinate uncertainty no >5 km and with no geospatial issues. To minimize spatial autocorrelation for our habitat suitability modeling, we spatially thinned the *L. delicata* occurrence records to 1 km using the “*thin*” function in the R package *spThin* (Aiello-Lammens et al. 2015), resulting in 424 occurrence points used for modeling.

### Species distribution modeling

We predicted the distribution of suitable *L. delicata* habitat using the Maximum Entropy species distribution modeling algorithm (i.e., MaxEnt; Phillips et al. 2006). Species distribution modeling explores the contrast between the environmental conditions of occupied sites (i.e., presence locations) and potentially unoccupied sites (i.e., locations of the environmental background) to estimate habitat suitability for a species across a landscape.

#### Defining the modeling domain

How the modeling domain is defined has considerable influence on model predictions (Elith et al. 2011; Merow et al. 2013). Our primary aim was to model the climatic constraints on the species’ distribution in the Upper Hunter Valley region and its immediate surrounds. We therefore set the modeling background as the extent of all biogeographic regions (Interim Biogeographic Regionalization for Australia (IBRA) v7.0) in this broad region (Supplementary Fig. S2).

#### Environmental variables

We pre-selected a set of 27 candidate predictor variables likely affecting the species’ distribution. These variables relate to climate, topography, hydrology, soil moisture, vegetation, and human influence (see Supplementary Table S1 for the full set of variables). We primarily included bioclimatic variables because, as an ectotherm, the distribution of *L. delicata* is likely influenced by temperature and precipitation regimes, as is the case for many ectothermic species (Araujo and Guisan 2006; Powney et al. 2010; Dervo et al. 2016). All environmental layers were downloaded at, or resampled to, a spatial resolution of ∼1 km^2^. All variables were resampled to fit the study background region using the “*mask*” function of the *raster* package (Hijmans et al. 2013). We then extracted environmental data from the locations of the thinned *L. delicata* occurrence points.

To reduce multicollinearity between variables, we first visually inspected the relationship of all predictor variables in the principle component (PC) environmental space (Supplementary Fig. S3). We then calculated the variance inflation factor (VIF) using the *“vifcor”* function of the *usdm* package (Naimi et al. 2014), which removes one variable with higher VIF from each pair of highly correlated variables (*r ≥* 0.7), retaining a final set of uncorrelated variables for model fitting: mean annual temperature (Bio1), mean diurnal range (Bio2), temperature seasonality (Bio4), precipitation of the driest month (Bio14), and precipitation seasonality (Bio15) (Supplementary Fig. S4). We used the *“ecospat.mantel.correlogram”* function in the *ecospat* package (Di Cola et al. 2017) to confirm that the spatial autocorrelation of occurrence records was not significantly different than 0 (Supplemntary Fig. S5).

#### Model fine-tuning and evaluation

MaxEnt model fine-tuning was achieved using the “*ENMevaluate*” function in the *ENMeval* package (v2.0.3; Muscarella et al. 2014; Kass et al. 2021), which runs MaxEnt across various combinations of feature classes and values of the regularization multiplier to enable comparisons of model performance. We then selected the MaxEnt settings that balanced model fit and predictive ability. First, we spatially partitioned the data using the *checkerboard2* cross-validation method to reduce the degree of overfitting (Kass et al. 2021). Models were then built with regularization multiplier options set to 1, 2, and 3 and with two different feature class combinations (1. linear; 2. linear + quadratic); this resulted in six parameter combinations. We sampled 10,000 background points (Phillips and Dudík 2008) across the modeling background.

The overall predictive performance of models was evaluated using the area under the curve (AUC) of the receiver operating characteristic (ROC) plot, which is a threshold-independent metric that determines the model’s ability to distinguish species occurrence records from background points (Hanley and McNeil 1982; Lobo et al. 2008). AUC values range from 0.5 (equivalent to that due to chance) to 1.0 (perfect performance), where values >0.7 indicate adequate accuracy and values >0.9 indicate very high predictive accuracy (Swets 1988). We constructed response curves of predictor variables from the best-fitting model to investigate how environmental predictors affected the predicted probability of habitat suitability by changing the variable of interest while holding the other variables at their mean. To quantify overfitting, we calculated the difference between training AUC and testing AUC (AUC_DIFF_). Overfit models generally perform well on training data but poorly on testing data, resulting in larger differences (Warren and Seifert 2011). Additionally, we calculated the omission rate for the 10th percentile presence (OM_10_), a common threshold-dependent metric for evaluating overfitting, where omission rates >10% typically indicate model overfitting (Radosavljevic and Anderson 2014). The model with the combination of parameters resulting in the lowest Akaike Information Criterion (corrected for small sample sizes; AICc) was selected as the best model (Warren and Seifert 2011; Muscarella et al. 2014).

### Measuring thermal preference of *L. delicata*

The set of body temperatures (*T*_b_s) selected by ectotherms in artificial thermal gradients (i.e., in the absence of ecological costs) is presumed to reflect their preferred body temperatures (Huey and Bennett 1987). To quantify the thermal preference range of *L. delicata*, individuals (*n* = 111) were placed into a 10 × 100 cm thigmo-thermal gradient constructed of aluminum. A near-linear thermal gradient ranging from 15 to 36°C was achieved by placing a cold plate beneath one end of the gradient and hanging two 250 W infrared ceramic heat lamps above the other end; such heat lamps eliminate the confounding effect of light on temperature selection (Sievert and Hutchinson 1988). Lizards were placed in the center of the gradient to initiate the experiment, and the locations of each lizard were monitored over a 2-h, 20-min period with video cameras positioned above the gradient. The first 20 mins of exploratory behavior were discarded. The entire gradient was cleaned with 70% ethanol between trials. A row of equally spaced iButtons spanning the length of the gradient was used to infer *T*_b_s at positions selected by lizards. This inference is justified given the short thermal time constant of small skinks such as *L. delicata* (1.30 ± 0.338 min; Fraser and Grigg 1984); thus, a *T*_b_ was only considered selected if a lizard remained inactive at a given position for at least 2 mins. These data were used to calculate the following thermal preference metrics: mean selected body temperature (*T*_set_), defined as the average body temperature selected within the thermal gradient; set-point range (*T*_set_; Hertz et al. 1993), defined as the central 50% of selected body temperatures (*T*_b_s) within the thermal gradient; and lower (*LT*_set_) and upper (*UT*_set_) bounds of the set-point temperatures (Barber and Crawford 1977). Experiments were performed on lizards in a post-absorptive state and conducted in a temperature-controlled room providing a background ambient temperature of 24°C; therefore, experiments were not influenced by varying ambient temperatures.

## Results

### *Lampropholis delicata* requires structurally complex habitat

*Lampropholis delicata* was present at all high-elevation sites on the Liverpool Range. The species was absent from all low-elevation sites (380–660 m a.s.l.) and from all but one mid-elevation site (780–840 m a.s.l.). The one mid-elevation site with *L. delicata* (664 m a.s.l.) was located at the lowest position of the Liverpool Range, which connects Coolah Tops to the eastern plateaux, indicating that the species is likely continuous across the Liverpool Range.

**Figure 2.**
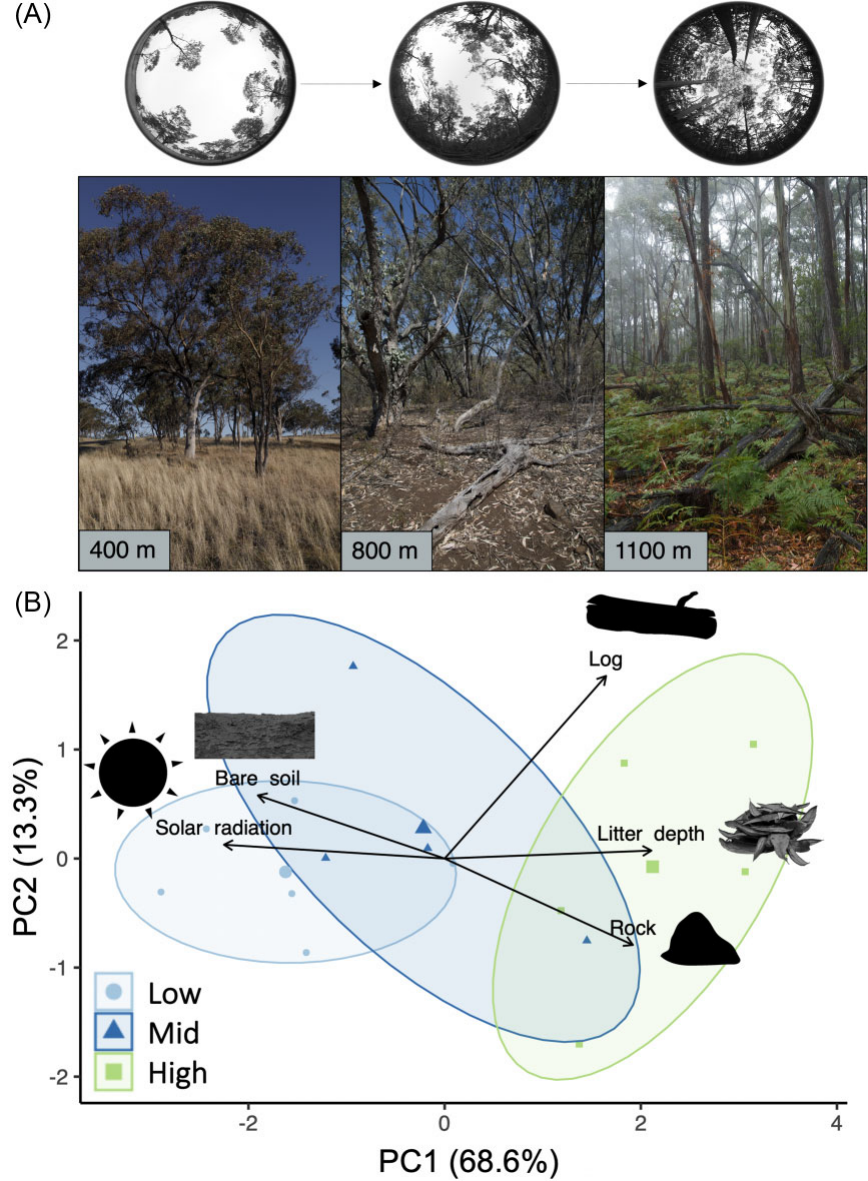
(A) Photographs showing examples of elevational variation in habitat structure in the study region, with canopy photographs taken at the same sites. (B) PCA ordination performed on the mean habitat structure variables at each site, with groups indicating broad elevational categories: 970–1070 m (high-elevation sites); 780–840 m (mid-elevation sites); and 380–660 m (low-elevation sites). Colored ovals around each group represent 95% confidence ellipses. Sites with *L. delicata* are indicated in the green ellipse. The first principle component (PC1) describes an increase in log cover, rock cover, and litter depth, and a decrease in bare soil and solar radiation.

The PCA of habitat structural attributes shows two components (PC1 and PC2) that explained a cumulative total of 81.9% of the variation in habitat structure among sites (Fig. 2). PC1 alone explained a considerable amount of variation (68.6%), with all five variables loading heavily onto this axis (Table 1). PC1 describes a gradient of increased logs, rocks, and litter depth, and reduced bare soil and solar radiation. PC2 explained 13.3% of the variation and describes a gradient of increased log cover. PC1 differed significantly among sites (*F*_2,12_ = 17.77, *P* < 0.001). Tukey’s post-hoc test revealed that habitat structure at low-elevation valley sites differed significantly from that of high-elevation sites (where *L. delicata* is found) in regards to PC1 (*P* = 0.003), whereas mid-elevation sites did not differ significantly from either low (*P* = 0.166) or high (*P* = 0.183) elevation sites.

**Table 1.** Loadings of the habitat structure variables for PC1 and PC2.

Variable	PC1	PC2
Rock cover (%)	**0.8**	**−**0.33
Log cover (%)	**0.68**	**0.7**
Bare soil cover (%)	**−0.79**	0.24
Litter depth (mm)	**0.88**	0.03
Solar radiation (%)	**−0.94**	0.05
Eigenvalue	3.43	0.66
% variance explained	68.6	13.3

The eigenvalues and percentage of variance explained are shown for each component. Values in bold represent variable loadings contributing the most to each axis (≥0.4).

### Deep leaf litter is thermally optimal at high-elevation *L. delicata* sites

**Figure 3.**
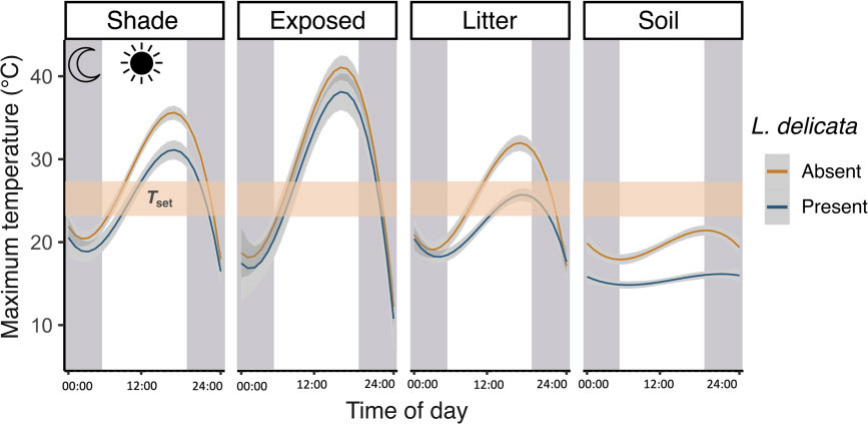
Hourly maximum microclimate temperatures recorded by the iButton data logger during October 2019. Thin blue and orange lines represent the line of best fit, bounded by gray-shaded 95% confidence intervals. The thermal set-point range (*T*_set_) of *L. delicata* is denoted by the orange horizontal band. Gray and white vertical zones represent night and day hours, respectively.

The mean thermal preference of *L. delicata* is 27.57°C (± 0.13). The lower (*LT*_set_ 25%) and upper (*LT*_set_ 75%) bounds of the set-point range (central 50% of selected temperatures) are 26.72 (± 0.15) and 28.4°C (± 0.13), respectively. Across all four microclimatic positions (shade, exposed, litter, and soil), temperatures are cooler at *L. delicata* presence sites compared to those of absence sites (Fig. 3). Shade and exposed microclimates exceed the species’ thermal preference at both presence and absence sites, whereas soil temperatures remain cooler than the thermal preference range. However, the thermal difference between presence and absence sites is most noteworthy in regards to litter temperatures; absence sites have litter temperatures that exceed the species’ thermal preference range at the hottest part of the day, whereas litter temperatures at presence sites remain within the species’ thermal preference range (Fig. 3). These thermal differences between presence and absence sites take place over an elevation gradient owing to the deeper litter layer and, hence cooler litter temperatures that characterize high-elevation *L. delicata* sites.

### Species distribution modeling

**Figure 4.**
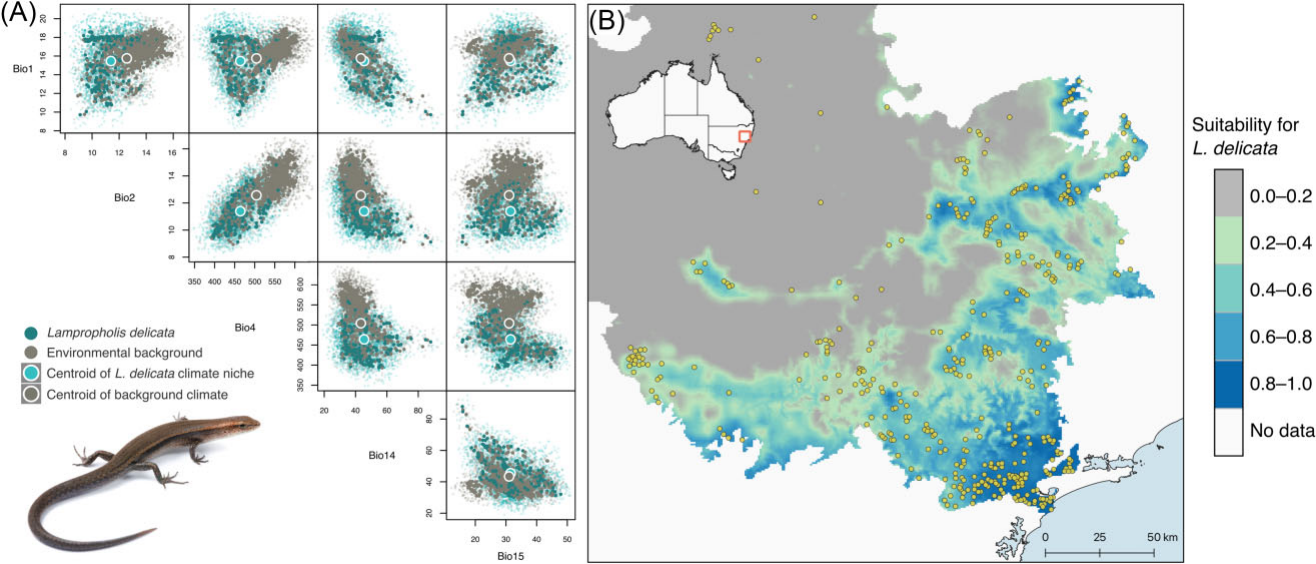
(A) Comparative scatter plots illustrating how the climate niche of *L. delicata* differs from the environmental background with respect to the five climatic variables used for modeling. (B) MaxEnt model prediction of environmentally suitable areas for the occurrence of *L. delicata* in the Hunter Valley region, NSW, Australia. Yellow points are records of the species and the gray–blue color gradient represents a continuous probability of habitat suitability with values ranging from 0 (unsuitable) to 1 (highly suitable).

The optimal model (i.e., with the lowest ΔAICc value) performed well with an AUC of 0.86 and included a regularization multiplier of 1 and LQ feature class. The difference between the training and testing AUC (i.e., AUC_DIFF_) was very low (0.012), indicating that the model predicts new data well and is not overfitted (Warren and Seifert 2011). The geographical prediction of the model is shown in Fig. 4. Model performance metrics of the six candidate models are provided in Supplementary Table S2.

The most important climatic predictor of the species’ distribution was temperature seasonality (bio4; 80% contribution), whereas the remaining four variables each contributed no more than 15% (Supplementary Fig. S6). Hence, the inland range limits of *L. delicata* in the Upper Hunter Valley region appear to be driven primarily by temperature seasonality, with a high probability of occurrence in areas with low temperature seasonality values (i.e., it requires more thermally stable areas). Response curves of the environmental variables from the Maxent model are provided in Supplementary Table S7.

## Discussion

Multiple factors may limit species’ distributions, and it is therefore necessary to investigate more than one suspected constraint imposed on species. We combined data on habitat structure, thermal physiology, microclimate, and macroclimate to understand the abiotic constraints that explain the inland range limit of the mesic-adapted skink *L. delicata*. To that end, populations occurring at the edge of a species’ distribution are invaluable study systems because they are the geographical point beyond which ecological conditions become unfavorable for persistence. Identifying these unfavorable conditions is therefore key to understanding species distributions and biogeographic patterns more generally.

In the Upper Hunter Valley region of NSW, dry valley habitats are structurally simple and lack essential microhabitats. *Lampropholis delicata* was only found at sites with structurally complex microhabitats at higher elevations (Fig. 2). This observation supports the hypothesized association between mesic ectotherms and habitats with a complex structural setting afforded by deep litter, logs, and rocks. Habitat structure has extensively been identified as a critical aspect of habitat selection in many reptiles because it mediates an individual’s ability to escape predators, reproduce, capture prey, avoid anthropogenic disturbances, and thermoregulate (Moermond 1979; Hecnar and M'Closkey 1998; Shine et al. 2002; Howard et al. 2003; Croak et al. 2008; Huey and Tewksbury 2009; Pike et al. 2010; Goulet et al. 2015). Among the aforementioned functions, the latter (thermoregulation) is likely the most pertinent limiting factor for *L. delicata* in our study region. Microhabitat structures such as litter and logs filter the environmental conditions, allowing lizards to access thermal regimes that are distinct from the broader regional climate (Woods et al. 2015). Deep leaf litter is a resource that *L. delicata* likely relies upon heavily as refuge from high temperatures. At the hottest part of the day (∼12:00–5:00 pm), when temperatures in both the shade and sun often exceed the species’ *T*_set_, litter temperatures still remain within the species’ *T*_set_ range, but only at sites with deep litter. Conversely, structurally simple lowland habitats (from which *L. delicata* is absent) have shallow leaf litter and hence poor thermal buffering capacity; temperatures at these sites exceed the species’ *T*_set_ at the hottest part of the day.

We suggest this is a contributing, if not major, explanation as to why the species is confined to structurally complex higher-elevation habitats at its inland range limits. The reasoning is as follows: Our thermal preference experiments show that individuals of *L. delicata* seek to behaviorally regulate *T*_b_s within a narrow *T*_set_ range (26.7–28.4°C). In many field studies of wild lizards, individuals often maintain *T*_b_s inside *T*_set_, despite the majority of the available environment tending away from the preferred range; this feat is achieved by exploiting favorable microclimatic variations provided by microhabitat structures (Adolph 1990; Hertz et al. 1993; Kearney and Predavec 2000; Muñoz and Losos 2018; Nordberg and Schwarzkopf 2019). However, in the present study, low-elevation habitats appear to offer no respite from unfavorably high temperatures (i.e., *T*_a_ > *T*_set_), even beneath the leaf litter, owing to their shallow depth at such sites. Thus, lizards must significantly increase their thermoregulatory effort in such environments, which typically leads to higher energetic costs, enhanced predation risk, and constrained activity times (Huey and Slatkin 1976; Neel and McBrayer 2018). It is the culmination of these costs, mediated via a decline in the availability of key habitat attributes over elevation, that may restrict the distribution of *L. delicata* at its inland range limit.

Our correlative SDM was constructed with biologically relevant climatic variables and is thus an implicit description of the realized climate niche of *L. delicata*. Projecting the modeled probability of occurrence into geographical space (Fig. 4) identified the position of the species’ inland range limits. As expected, areas that are more inland (western) and low in elevation have a lower probability of occurrence for the species; this includes the dry, open lowlands of the Upper Hunter Valley. The refugial populations of *L. delicata* on the Liverpool Range diverged from nearby populations during the early to mid Pliocene (Chapple et al. 2011), suggesting that the dry, low-elevation habitat surrounding the Liverpool Range (i.e., the Upper Hunter Valley) has been a major dispersal barrier for the species in the past. While the precise paleoclimatic factors that drove vicariance in this region remain unknown, the present study supports the notion that the Upper Hunter Valley is a dispersal barrier for *L. delicata* under contemporary climate and implicates the Liverpool Range as refugial habitat.

High temperature seasonality has previously been identified as a primary factor driving range limits in some lizards (Vera et al. 2023), frogs (Wiens et al. 2006), birds (Sanín and Anderson 2018), mammals (Frey et al. 2013), and plants (Gong et al. 2020; Liao et al. 2021). High temperature seasonality is the macroclimatic factor that appears to be defining the present inland range limits of *L. delicata*. At the edge of the species’ distribution, high elevations of the Liverpool Range provide thermally stable refugia in an otherwise highly seasonal region. Indeed, our surveys found populations of *L. delicata* occurring along the Liverpool Range, from Coolah Tops to Crawney Pass National Park, all of which belong to the same genetic subclade (McDiarmid et al., submitted for publication), suggesting reliable population connectivity along refugial habitats of the Liverpool Range.

When mesic species distributions fragment during climatically restrictive periods, they likely do so not strictly because of shifts in macroclimates but also because macroclimatic shifts drive changes in vegetation community (and hence habitat structures), which, in turn, alter the availability of microclimates (Lembrechts and Nijs 2020). Understanding species range limits thus requires an understanding of the inextricable link between macroclimate, habitat structure, and microclimate. We posit that *L. delicata*, and possibly most other mesic ectotherms, can persist in areas in which (a) the macroclimate regime is within its physiological tolerances and/or (b) individuals have access to microclimates that buffer against an unfavorable regional climate.

## Conclusion

In a mesic-adapted lizard occurring at its inland range limits, we have shown how measured data on habitat structure and microclimate can add another dimension to studies of species’ range limits. Simply modeling a species’ distribution with SDMs can be of great use for most purposes, as it tells us where a species is likely to occur and identifies the variables associated with its occurrence. However, this approach alone cannot elucidate the finer-scale ecological processes that organisms actually experience on the ground. Our approach enabled us to empirically describe the transition in habitat and microclimatic at both presence and absence sites at a species’ range limit. When these microclimatic data are compared with a species’ thermal preference data, it provides a more direct understanding of how ecophysiology contributes to the range limits we observe.

## Animal ethics

This research was conducted in accordance with appropriate collection and research permits (New South Wales: SL102248, Victoria: 10009231) and was approved by the Monash University Animal Ethics Committee (18055, BSCI/2018/09).

## Supplementary Material

icad124_Supplemental_File

## Data Availability

The occurrence data, habitat data, and environmental variables used in the SDM are available from the authors upon request.
