# Supplementary material for: Identifying the abiotic factors that determine the inland range limits of a mesic-adapted lizard species
Source: Integr Comp Biol. 2023 Oct 19;64(1):55–66. doi: 10.1093/icb/icad124 (PMC11277862; doi:10.1093/icb/icad124)
Supplement: icad124_Supplemental_File [file icad124_supplemental_file.docx]

**SUPPORTING INFORMATION FILE 1**

**Identifying the abiotic factors that determine the inland range limits of a mesic-adapted lizard species**

Jules E. Farquhar, Wyn Russell, David G. Chapple

*Integrative and Comparative Biology*

**S1 Additional information on iButtons deployment**

We measured the thermal regimes of different microclimates at each site using iButtons (see 2.3 of Methods). We focused on leaf litter as the primary shelter site for *Lampropholis delicata* because (1) the species frequently shelters within litter and (2) it is the most abundant shelter type; leaf litter was present at all sites, whereas other buffering microhabitats, such as rocks and logs, were not present at all sites. At all sites, we placed iButton data logger stations (Figure S1) in the deepest section of leaf litter available at the site, hence the litter temperature measurements are expected to represent the most thermally buffered microclimates available for skinks to avoid unfavourable ambient temperatures at each site. The depth of leaf litter above the iButtons was intentionally non-standardised, as litter depths tend to be shallower at drier, low-elevations. Manipulating litter depth to be standard would not reflect natural differences in the thermal buffer provided by leaf litter over an elevational cline, which is expected to be a relevant range-limiting factor. The exposed and soil iButtons were placed inside deflated balloons so as to prevent water from destroying the data loggers (Figure S1).


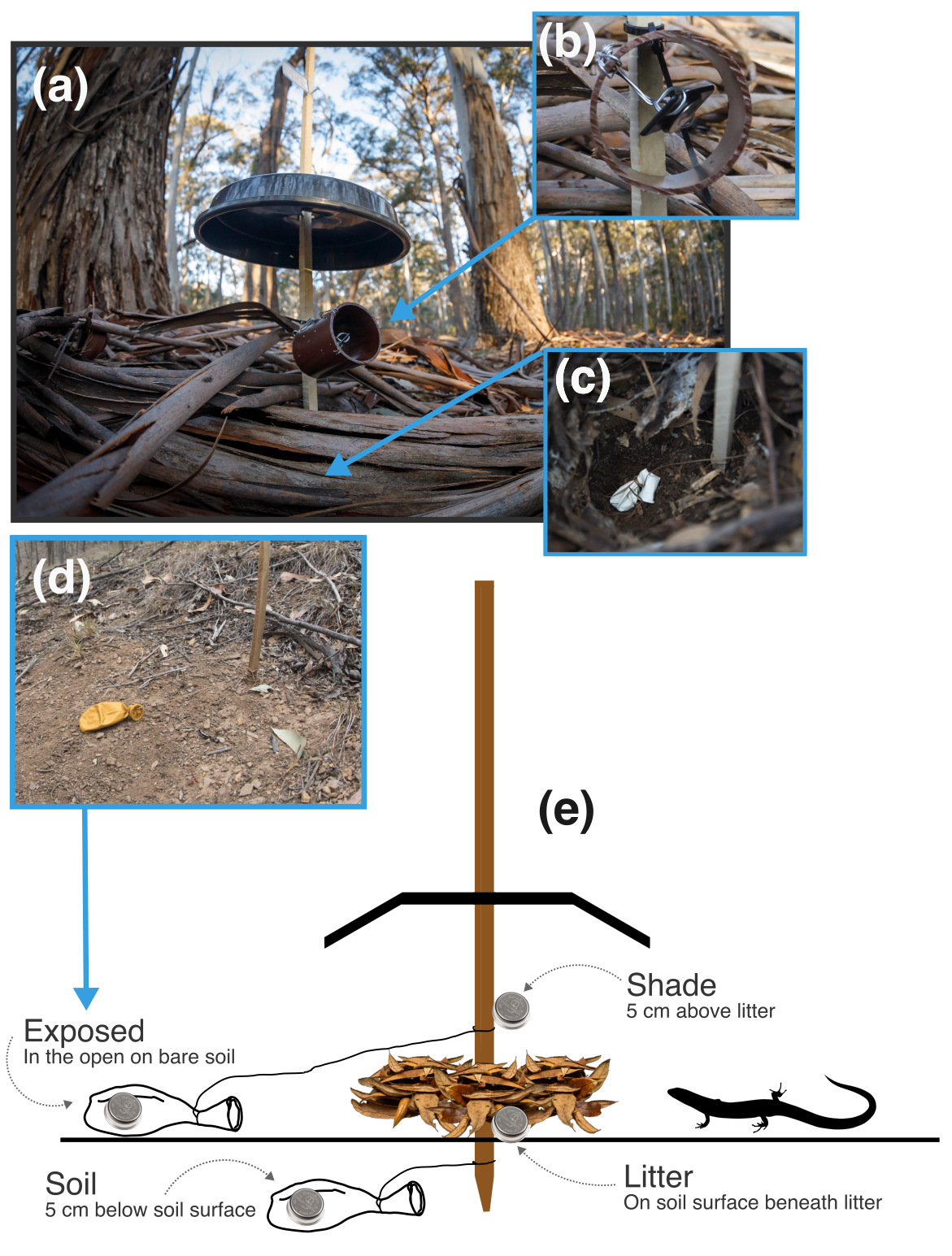


Figure S1. (a) Example iButton data logger station setup. (b) View inside the PVA tube showing how the shade iButton is attached to a plastic fob which is then suspended from the roof using wire, so as to avoid direct contact with the walls; (c) soil iButton inside a balloon before being buried beneath the soil and litter; (d) exposed iButton inside a balloon on bare soil and unshaded; (e) diagrammatic example of a site’s entire iButton arrangement. These four iButton positions capture the thermal regimes of four different microhabitats accessed by *Lampropholis delicata*.

**S2. Species distribution modelling**

Figure S2. The modelling background IBRA bioregions with occurrence points included.

Table S1. Full set of spatial layers considered in variable selection for modelling the distribution of *Lampropholis delicata* using the maxent algorithm. Bold indicates the database and a URL is provided. Italics indicate derived data.

| Source/layer | Year | Spatial Resolution |
| --- | --- | --- |
| **WorldClim v2.1** (http://www.worldclim.org/) |  |  |
| BO1 Annual Mean Temp (˚C) | 1970–2000 | 1 x 1 km |
| BIO2 Mean Diurnal Range (˚C) | 1970–2000 | 1 x 1 km |
| BIO3 Isothermality (100 * BIO2 / BIO7) | 1970–2000 | 1 x 1 km |
| BIO4 Temp Seasonality (100 * SD) | 1970–2000 | 1 x 1 km |
| BIO5 Max Temp of Warmest Month (˚C) | 1970–2000 | 1 x 1 km |
| BIO6 Min Temp of Coldest Month (˚C) | 1970–2000 | 1 x 1 km |
| BIO7 Temp Annual Range (˚C) (BIO5–BIO6) | 1970–2000 | 1 x 1 km |
| BIO8 Mean Temp of Wettest Quarter (˚C) | 1970–2000 | 1 x 1 km |
| BIO9 Mean Temp of Driest Quarter (˚C) | 1970–2000 | 1 x 1 km |
| BIO10 Mean Temp of Warmest Quarter (˚C) | 1970–2000 | 1 x 1 km |
| BIO11 Mean Temp of Coldest Quarter (˚C) | 1970–2000 | 1 x 1 km |
| BIO12 Annual Precip (mm) | 1970–2000 | 1 x 1 km |
| BIO13 Precip of Wettest Month (mm) | 1970–2000 | 1 x 1 km |
| BIO14 Precip of Driest Month (mm) | 1970–2000 | 1 x 1 km |
| BIO15 Precip Seasonality (CV) | 1970–2000 | 1 x 1 km |
| BIO16 Precip of Wettest Quarter (mm) | 1970–2000 | 1 x 1 km |
| BIO17 Precip of Driest Quarter (mm) | 1970–2000 | 1 x 1 km |
| BIO18 Precip of Warmest Quarter (mm) | 1970–2000 | 1 x 1 km |
| BIO19 Precip of Coldest Quarter (mm) | 1970–2000 | 1 x 1 km |
| **ENVIREM** (https://envirem.github.io/) |  |  |
| Thornthwaite aridity index | 1960–1990 | 1 x 1 km |
| tri — terrain roughness index | NA | 1 x 1 km |
| topoWet — SAGA-GIS topographic wetness index | NA | 1 x 1 km |
| **Geoscience Australia** (http://www.ga.gov.au/search/index.html#/) |  |  |
| *Euclidean Distance to Water Courses* | NA | 1 x 1 km |
| *Euclidean Distance to Water Bodies* | NA | 1 x 1 km |
| **Terrestrial Ecosystem Research Network (TERN)** (https://www.tern.org.au/) |  |  |
| Total Available Soil Water (mm) | in February, 2022 | 1 x 1 km |
| **NASA Earthdata** (https://earthdata.nasa.gov/) |  |  |
| Normalized Difference Vegetation Index (NDVI) (MOD13A3) | in December, 2000–2021 | 1 x 1 km |
| **Socioeconomic Data and Applications Center (sedac)** (https://sedac.ciesin.columbia.edu/) |  |  |
| Global Human Influence Index (Geographic) v2 | 1995–2004 | 1 x 1 km |


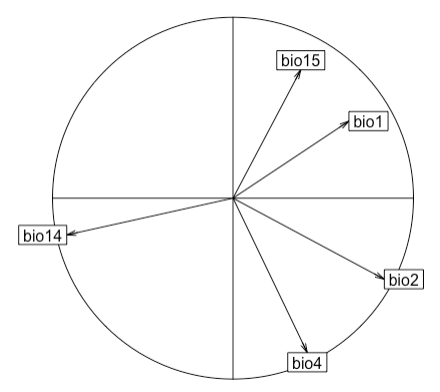

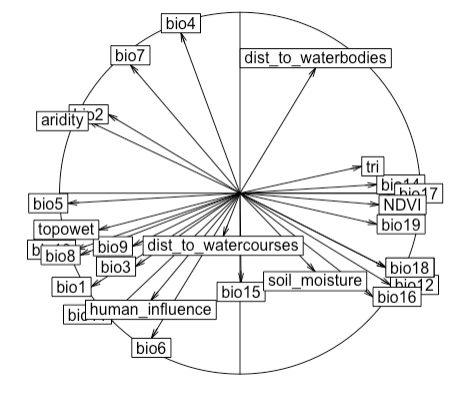


A

B

Figure S3. PCA biplots showing correlations among variables for (A) the full set of variables and (B) the final set of variables used for habitat suitability modelling.


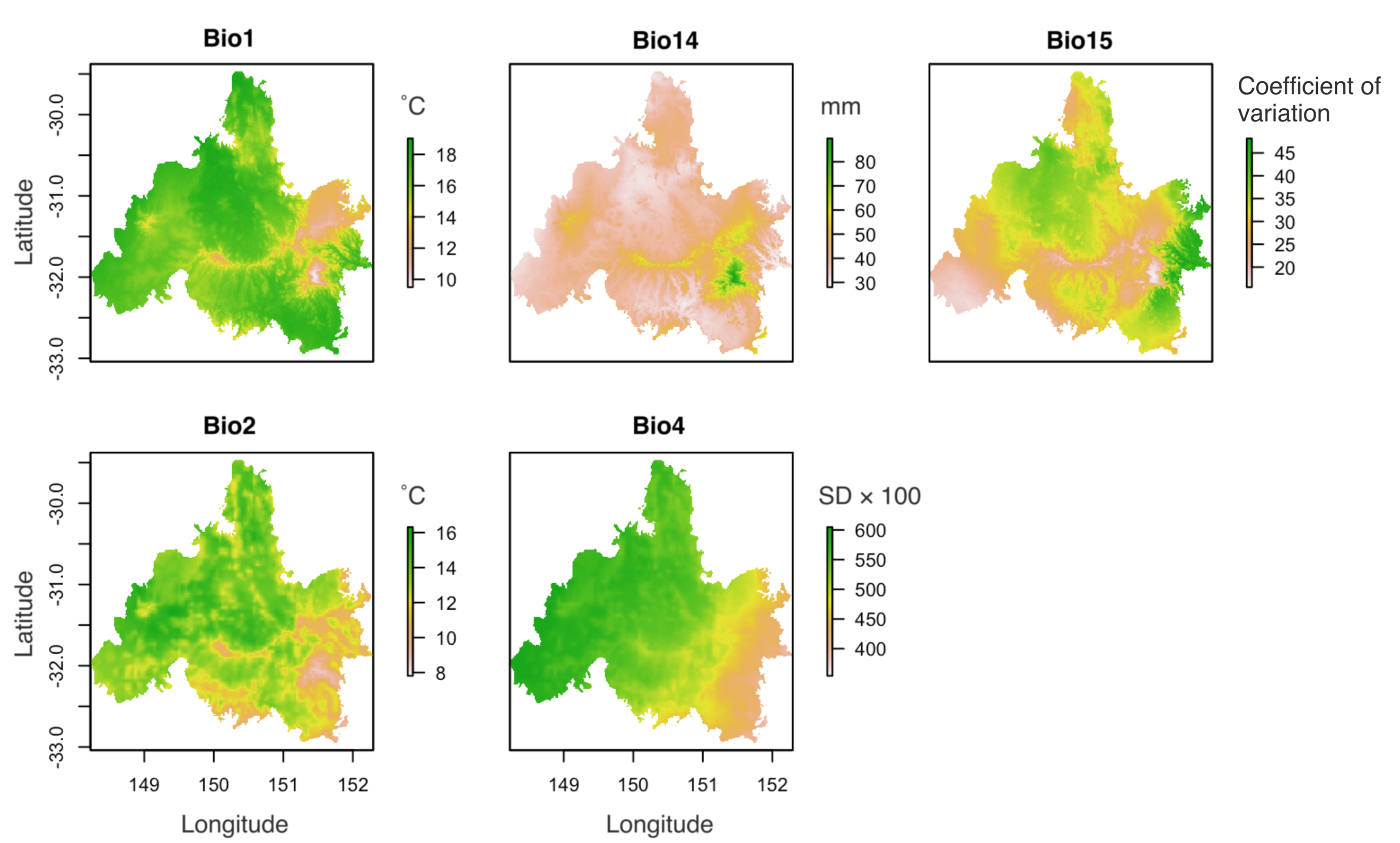


Figure S4. Maps showing the range of environmental variation across the modelling domain for each of the final predictor variables used in habitat suitability modelling. **Bio1** = Annual Mean Temperature; **Bio2** = Mean Diurnal Range (Mean of monthly (max temp - min temp)); **Bio4** = Temperature Seasonality (standard deviation ×100); **Bio14** = Precipitation of Driest Month; **Bio15** = Precipitation Seasonality (Coefficient of Variation)


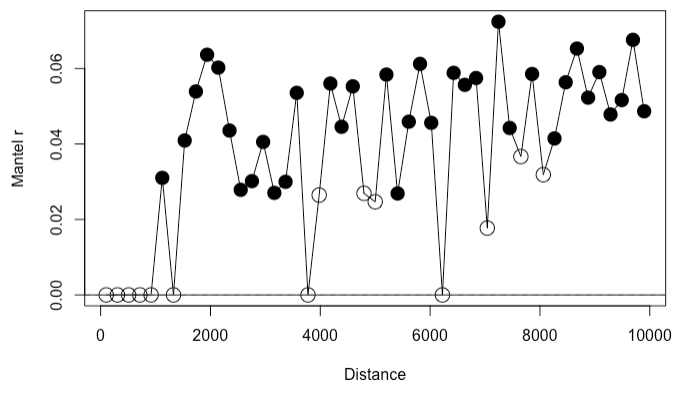
Figure S5. Test for spatial autocorrelation using the *‘ecospat.mantel.correlogram’* function in the *ecospat* package (Di Cola et al., 2017). The Mantel r value shows that spatial autocorrelation of occurrence records was not significantly different than 0 at most distances. This is because we spatially thinned our occurrence data by 1 km.

Table S2. Model performance evaluation metrics of the MaxEnt model generated using ENMeval (Muscarella et al., 2014).

| Model No. | RM | FC | AUC_TRAIN_ | AUC_DIFF_ | OR_10_ | OR_MTP_ | ΔAICc |
| --- | --- | --- | --- | --- | --- | --- | --- |
| 1 | 1 | LQ | 0.8638244 | 0.0120507 | 0.09916141 | 0.00510204 | 0 |
| 2 | 2 | LQ | 0.86251726 | 0.01259055 | 0.09918718 | 0.00510204 | 5.98196359 |
| 3 | 3 | LQ | 0.86241774 | 0.01282648 | 0.10123636 | 0.00510204 | 7.01704817 |
| 4 | 1 | L | 0.86123226 | 0.01268696 | 0.09931295 | 0.00510204 | 17.9083044 |
| 5 | 2 | L | 0.86127988 | 0.0129325 | 0.09931295 | 0.00510204 | 20.488973 |
| 6 | 3 | L | 0.86126845 | 0.01307027 | 0.1017882 | 0.00510204 | 23.7031728 |

Figure S6. Estimates of relative (%) contributions of environmental predictor variables to the Maxent model.

Figure S7. Response curves characterizing how each environmental variable affected the MaxEnt predictions for the best performing model.

**S3 *Lampropholis delicata collection and husbandry***

Delicate skinks were collected from four locations: 60 lizards from Coolah Tops National Park (30 from each of two sites spaced 5 km apart) and 59 lizards from two eastern locations where mountain populations become contiguous with the Great Dividing Range at large (30 from Crawney Pass National Park and 29 from Hanging Rock State Forest). Only adult (SVL > 34 mm), full-tailed (tail length > SVL) male lizards were collected so as to avoid the potential confounding effects of gravidity and tail loss on physiological performance (Shine 2003; Cromie and Chapple 2012). Lizards were transported to Monash University (Clayton, Victoria, Australia) for physiological experiments and, on arrival, individuals received a Visual Implant Elastomer (VIE, Northwest Marine Technology, WA) for identification. Lizards were housed in groups of six individuals within large plastic containers (300 × 230 × 370 mm). A small plastic pot and two terracotta tiles were added to provide shelter. UV lighting was activated above each container from 0800 to 1800 h. All housing containers were located in a temperature-controlled room with an ambient temperature of 22–23 °C and room lighting activated from 0600 to 2000 h. A timer-controlled heat mat under one end of each container was set to 32 °C to create a thermal gradient of 22–32 °C during the day. Lizards were fed crickets (*Acheta domesticus*), dusted with vitamin supplement (Reptivite^TM^), three times weekly and provided water *ad libitum*.
